# Factors associated with same-sex experience in people with non-psychotic mental disorders and suicidal ideation

**DOI:** 10.1192/j.eurpsy.2021.466

**Published:** 2021-08-13

**Authors:** M. Zinchuk, M. Beghi, E. Beghi, G. Kustov, E. Pashnin, N. Voinova, A. Avedisova, A. Guekht

**Affiliations:** 1 Suicide Research And Prevention, Moscow Research and Clinical Center for Neuropsychiatry, Moscow, Russian Federation; 2 Department Of Mental Health, AUSL Romagna, Cesena, Italy; 3 Laboratory Of Neurological Disorders, Istituto di Ricerche Farmacologiche Mario Negri IRCCS, Milan, Italy

**Keywords:** Suicide, NSSI, LGBT, non-psychotic mental disorders

## Abstract

**Introduction:**

People with mental disorders who had same-sex experience (SSE) are at increased risk of self-injurious behavior probably due to the double stigma phenomenon, which severity varies in different societies. So far, there is a knowledge gap on factors associated with SSE in Russian psychiatric patients.

**Objectives:**

We aimed to investigate variables associated with homosexual experience in Russian patients with non-psychotic mental disorders (NPMD) and suicidal ideation (SI).

**Methods:**

In a case-control study (1:1.5): 92 female patients with NPMD and SI with lifetime SSE were compared with 138 patients without homosexual experience. All patients underwent a psychiatric examination, Self-Injurious Thoughts and Behaviors Interview (Nock MK, 2007) and semi-structured interview to assess demographic, clinical, and behavioral features. Mann-Whitney, Fishers exact test and Pearson’s chi-squared were used as statistical methods.

**Results:**

Groups did not differ in education level, marital status, family history of suicidal behavior, traumatic events exposure and lifetime eating disorders (all:p>0.05). More patients with SSE had family history of non-suicidal self-injuries (NSSI), were dissatisfied with their parenting style, had a higher number of unprotected sexual contacts with unfamiliar persons, practiced group sex, had a history of sexual abuse, illicit drug use experience, were smokers, had piercing and severe body modifications. Lifetime history of suicide plan, attempts and NSSI were significantly more common in people with SSE (all:<0.05).

**Conclusions:**

A number of suicide risk factors were found to be more prevalent in people with SSE. Homosexual experience in people with mental disorders is associated with an increased risk of NSSI, suicide plan development and suicide attempts.

**Disclosure:**

No significant relationships.

